# Integrin-Mediated TIMP1 Signaling Reprograms Liver Macrophages and Accelerates Colorectal Cancer Metastasis

**DOI:** 10.3390/cells15010029

**Published:** 2025-12-23

**Authors:** Jialiang Liu, Liming Zhao, Lin Wang, Guoli Sheng, Pu Cheng, Mingyu Han, Guoxin Li, Zhaoxu Zheng

**Affiliations:** 1 Department of Colorectal Surgery, National Cancer Center/National Clinical Research Center for Cancer/Cancer Hospital, Chinese Academy of Medical Sciences and Peking Union Medical College, Beijing 100020, China; 2 Cancer Center, Beijing Tsinghua Changgung Hospital, School of Clinical Medicine, Tsinghua Medicine, Tsinghua University, Beijing 102218, China; 3 The State Key Laboratory of Molecular Oncology, National Cancer Center/National Clinical Research Center for Cancer/Cancer Hospital, Chinese Academy of Medical Sciences and Peking Union Medical College, Beijing 100020, China; 4 Department of Urology, National Cancer Center/National Clinical Research Center for Cancer/Cancer Hospital, Chinese Academy of Medical Sciences and Peking Union Medical College, Beijing 100020, China; 5 Department of Hepatobiliary Surgery, National Cancer Center/National Clinical Research Center for Cancer/Cancer Hospital, Chinese Academy of Medical Sciences and Peking Union Medical College, Beijing 100020, China

**Keywords:** colorectal cancer, liver metastasis, TIMP1, M2 macrophage polarization, pre-metastatic niche

## Abstract

Background: Colorectal cancer (CRC) frequently metastasizes to the liver (CRLM), where M2-polarized macrophages shape an immunosuppressive pre-metastatic niche. The molecular cues driving this polarization remain unclear. Methods and Results: Using integrated transcriptomics, patient cohorts, and mouse models, we investigated the role of tissue inhibitor of metalloproteinases-1 (TIMP1) in CRLM. TIMP1 was consistently overexpressed in CRC tissues and associated with poor overall survival. CRC cells secreted TIMP1 into the tumor microenvironment, where it induced M2-like macrophage polarization and increased the expression of immunosuppressive mediators such as CSF1 and IRF4. In vivo, TIMP1 overexpression enhanced, whereas its knockdown reduced, liver metastatic burden. Immune profiling and depletion experiments indicated that these pro-metastatic effects were largely macrophage-dependent. Mechanistically, TIMP1 engaged CD63/β1-integrin on macrophages, activating AKT/mTOR signaling and stabilizing the M2 phenotype. Conclusions: CRC-derived TIMP1 remodels liver macrophages via the CD63/β1-integrin–AKT/mTOR pathway to promote a hepatic pre-metastatic niche. Pharmacologic inhibition of this signaling axis with the integrin antagonist cilengitide suppressed macrophage M2 markers and liver colonization in mice, supporting TIMP1–integrin signaling as a potential therapeutic target.

## 1. Introduction

Colorectal cancer (CRC) remains one of the most prevalent and lethal malignancies worldwide, ranking as the third most common cancer and the second leading cause of cancer-related death globally [1]. A major contributor to CRC mortality is liver metastasis (CRLM), which occurs in nearly 50% of advanced-stage CRC patients and is often the direct cause of death [2,3,4]. Despite improvements in systemic therapies and surgical interventions, the five-year survival rate for patients with CRLM remains dismal, underscoring the urgent need to better understand the molecular mechanisms driving metastatic progression and to identify new therapeutic targets [5,6].

Mounting evidence suggests that the pre-metastatic niche (PMN), a permissive microenvironment established in distant organs before the arrival of circulating tumor cells, plays a pivotal role in facilitating metastasis [7,8]. The hepatic PMN is orchestrated by tumor-derived soluble factors that remodel the extracellular matrix (ECM), alter vascular permeability, and recruit immunosuppressive cells to create a favorable metastatic milieu [9,10,11]. Among these immune components, tumor-associated macrophages (TAMs), particularly those with an M2-like phenotype, have been identified as key mediators of immune evasion, angiogenesis, and metastatic colonization [12,13]. However, the upstream signals and regulatory molecules responsible for macrophage polarization within the CRC liver metastatic niche remain incompletely defined.

Tissue inhibitor of metalloproteinases-1 (TIMP1), classically known for its inhibitory effects on matrix metalloproteinases (MMPs), has recently emerged as a multifunctional protein implicated in cancer progression [14,15]. While initially considered a tumor suppressor due to its anti-proteolytic activity, TIMP1 is now recognized to promote tumorigenesis by regulating cell proliferation, apoptosis resistance, and metastasis through MMP-independent pathways [16]. However, the precise role of TIMP1 in shaping the immune microenvironment—particularly in regulating macrophage polarization and PMN formation during CRLM—remains poorly understood.

In this study, we asked whether CRC-secreted TIMP1 drives liver metastasis by reprogramming hepatic macrophages through CD63/β1-integrin–AKT/mTOR signaling. We addressed this question by combining analyses of patient datasets with cell-based and mouse models in which TIMP1 expression was either increased or reduced. Our data indicate that TIMP1 is a macrophage-dependent driver of CRC liver metastasis, promoting M2 polarization, pre-metastatic niche formation, and metastatic colonization. Finally, we examined whether pharmacologic blockade of integrin signaling can restrain TIMP1-induced macrophage reprogramming and liver metastasis.

## 2. Materials and Methods

### 2.1. Cell Culture and Treatment

The human colorectal cancer (CRC) cell lines SW480 and HCT116, the murine CRC cell lines CT26 and MC38, the murine macrophage cell line RAW 264.7, and the human monocytic leukemia cell line THP-1 were all obtained from the Cell Bank of the Chinese Academy of Sciences (Shanghai, China). HCT116, CT26, and MC38 cells were cultured in RPMI 1640 medium (Gibco, Grand Island, NY, USA) supplemented with 10% fetal bovine serum (FBS), while SW480 and THP-1 cells were cultured in DMEM medium (Gibco, Grand Island, NY, USA) containing 10% FBS. All cells were maintained at 37 °C in a humidified atmosphere with 5% CO_2_. To induce differentiation of THP-1 cells into macrophages, cells were treated with 100 ng/mL phorbol 12-myristate 13-acetate (PMA) (Sigma-Aldrich, St. Louis, MO, USA) for 48 h, followed by replacement with fresh PMA-free medium and further cultured for 24 h to obtain a stably differentiated macrophage phenotype.

### 2.2. Vector and the Establishment of Overexpression and Knockdown Cell Lines

TIMP1 cDNA was amplified by RT-PCR and cloned into the pLVX-Puro vector. After sequence verification, the plasmid was co-transfected with psPAX2 and pMD2.G into HEK293T cells to produce lentiviral particles. Viral supernatants were collected 48 h post-transfection, filtered through a 0.45 μm filter, and mixed with 1 μg/mL polybrene (Sigma-Aldrich, St. Louis, MO, USA) to infect target cells for 24 h. Subsequently, cells were selected with 1 mg/mL puromycin (Sigma-Aldrich, St. Louis, MO, USA) for 7 days. Short hairpin RNAs (shRNAs) (Table S1) were synthesized by GenePharma (Suzhou, China) and transfected into cells using Lipofectamine 2000 (Invitrogen, Thermo Fisher Scientific Inc., Waltham, MA, USA) according to the manufacturer’s instructions, with transfection lasting 48 h. Small interfering RNAs (siRNAs) and the corresponding negative control siRNA scramble (si-NC) used in this study were purchased from Shanghai Qingke Biotechnology Co. (Shanghai, China). The siRNA sequences are detailed in Supplementary Table S2. Cells were cultured until 70–80% confluence and then transfected with siRNAs using Lipofectamine 3000 Transfection Reagent (Invitrogen, Thermo Fisher Scientific, Inc. Waltham, MA, USA). After 48 h of incubation, transfected cells were harvested for subsequent experiments. Knockdown efficiency was determined by RT-qPCR or Western blot analysis following siRNA transfection.

### 2.3. GEO Data Processing

We downloaded the GSE127069, GSE54986, GSE137511, and GSE14297 datasets from the GEO database in MINiML format. To ensure data consistency and comparability, quantile normalization was performed using the normalize.quantiles function from the preprocessCore R package (version 1.64.0). For datasets generated on the same platform but from different batches, batch effects were removed using the removeBatchEffect function from the limma package to reduce technical bias. The effectiveness of normalization was evaluated with boxplots, while the removal of batch effects was assessed by comparing principal component analysis (PCA) plots before and after correction (Supplementary Figure S1A–L).

### 2.4. TCGA and GTEx Data Processing

RNA-seq STAR-count data and associated clinical information for colorectal tumors were downloaded from the TCGA database (https://portal.gdc.cancer.gov; accessed on 15 March 2024). Transcript per million (TPM) values were extracted and transformed using log2(TPM + 1) normalization. A total of 620 samples with both RNA-seq data and clinical information were retained for subsequent analyses. GTEx data (version V8) were also utilized, with further details available on its official website (https://gtexportal.org/home/datasets; accessed on 15 March 2024). All statistical analyses were performed using R software (v4.0.3; Foundation for Statistical Computing, Vienna, Austria).

### 2.5. CCLE Data Processing

The gene expression matrix for CRC tumor cell lines was obtained from the CCLE dataset (https://depmap.org/portal/data_page/?tab=allData; accessed on 15 March 2024). Statistical analyses were conducted using R software (v4.0.3).

### 2.6. Quantitative Real-Time PCR (qRT-PCR)

Total RNA was extracted using TRIzol reagent (Invitrogen, Thermo Fisher Scientific Inc., USA), and its purity was assessed by NanoDrop spectrophotometry (NanoDrop 2000, Thermo Fisher Scientific Inc., USA) with an A260/A280 ratio of 1.8–2.0. Reverse transcription and genomic DNA removal were performed with the PrimeScript RT kit (Takara Bio, Shiga, Japan) according to the manufacturer’s instructions (37 °C for 15 min, 85 °C for 5 s). The PCR reaction mixture (20 μL) consisted of 10 μL of SYBR Green Mix, 0.4 μL of forward and reverse primers (10 μM), 2 μL of cDNA, and 7.2 μL of RNase-free water. Amplification was conducted using the Applied Biosystems 7500 Fast system (Applied Biosystems, Thermo Fisher Scientific Inc., USA) under the following cycling conditions: 95 °C for 30 s, followed by 40 cycles of 95 °C for 5 s and 60 °C for 30 s. Melting curve analysis was performed to verify specificity. All samples were analyzed in triplicate. Primers were synthesized by Sangon Biotech (Shanghai, China) and are listed in Table S3. Relative mRNA expression was calculated using the 2^−ΔΔCt^ method, with GAPDH serving as an internal control.

### 2.7. Western Blot Analysis

Cells were lysed in RIPA buffer (Beyotime Biotechnology, Shanghai, China) containing protease inhibitors (Roche Diagnostics, Basel, Switzerland). Protein concentrations were measured using the BCA assay kit (Thermo Fisher Scientific Inc., Waltham, MA, USA). Equal amounts of protein (30–50 μg) were separated by 10% SDS-PAGE and transferred to PVDF membranes (Millipore, Merck KGaA, Darmstadt, Germany) at 250 mA for 90 min. Membranes were blocked with 5% nonfat milk in TBST for 1 h, then incubated overnight at 4 °C with primary antibodies (Table S4). The next day, membranes were washed three times with TBST (10 min per wash) and incubated for 1 h at room temperature with HRP-conjugated secondary antibodies. Protein bands were visualized using an ECL detection kit, imaged on an iBright FL1500 (Invitrogen, Thermo Fisher Scientific Inc., Waltham, MA, USA), and quantified with ImageJ software (version 1.53; National Institutes of Health, Bethesda, MD, USA). GAPDH was used as a loading control.

### 2.8. Co-Immunoprecipitation (Co-IP)

Cell lysates were precleared and then incubated overnight at 4 °C with protein A/G agarose beads (Santa Cruz Biotechnology, Dallas, TX, USA) conjugated with either normal IgG or anti-TIMP1 antibodies. After washing with lysis buffer, bound proteins were eluted and analyzed by Western blotting.

### 2.9. Hematoxylin and Eosin (H&E) Staining

Mouse liver tissues were fixed overnight in 10% buffered formalin, embedded in paraffin, and sectioned at 4 μm. Sections were mounted on slides, deparaffinized, rehydrated through graded alcohols, and stained with hematoxylin and eosin to evaluate histomorphology.

### 2.10. Tissue Microarray (TMA) Construction and Immunohistochemistry (IHC)

Representative tumor regions were selected from formalin-fixed paraffin-embedded (FFPE) blocks of each patient, and tissue microarrays (TMAs) were constructed using a tissue arrayer (Beecher Instruments, Sun Prairie, WI, USA). TMA sections (4 μm thick) were subjected to IHC staining using primary anti-TIMP1 antibodies (1:1000 dilution). DAB (Abcam, Cambridge, UK) was used for signal detection, and hematoxylin was used for counterstaining. Slides were scanned using a PANNORAMIC MIDI digital scanner (3DHISTECH Ltd., Budapest, Hungary) to obtain high-resolution images. All slides were independently reviewed by two to three pathologists. Staining intensity was evaluated using the H-score method.

### 2.11. Immunofluorescence Staining of Mouse Liver

Paraffin sections of mouse liver were deparaffinized in xylene and sequentially rehydrated through graded ethanol series (100%, 100%, 90%, 80%, 70%) followed by double-distilled H_2_O. Antigen retrieval was performed by boiling sections in 0.01 M sodium citrate buffer (pH 6.0) for 20 min. Sections were blocked for 1 h in blocking buffer (PBS containing 5% normal donkey serum, 1% BSA, 2% cold-water fish skin gelatin, and 0.1% Triton X-100), then incubated with primary antibodies (CD206 1:200, F4/80 1:300) at 4 °C for 12 h. After washing, sections were incubated with species-matched fluorescent secondary antibodies (anti-rabbit and anti-mouse) for 1 h at room temperature. Following washes, nuclei were counterstained with DAPI (1 μg/mL). After final washes, sections were mounted with antifade mounting medium and imaged using an LSM880 confocal microscope (Zeiss, Jena, Germany). Fluorescence signal intensity was analyzed with ZEN software (Zeiss, Jena, Germany).

### 2.12. Cellular Immunofluorescence

RAW and THP-1 cells were seeded on glass coverslips. When cells reached ~70% confluency, immunofluorescence staining was performed. Cells were fixed with 4% paraformaldehyde for 15 min, washed with PBS, and permeabilized with 0.1% Triton X-100 for 10 min. After blocking with blocking buffer (OriGene Technologies, ZLI-0956, Beijing, China) at room temperature for 1 h, cells were incubated with primary antibodies overnight at 4 °C. Following washes, cells were incubated with fluorescently labeled secondary antibodies for 1 h, counterstained with DAPI, and imaged using a confocal microscope (Olympus FV3000, Olympus Corporation, Tokyo, Japan). Recombinant human IL-4 and IL-13 were added 48 h prior to cell harvesting.

### 2.13. Preparation of Tumor-Conditioned Medium (CM)

MC38 and CT26 cells were maintained in complete DMEM supplemented with 10% FBS, 1% penicillin/streptomycin, 1% non-essential amino acids, 1% sodium pyruvate, 1% HEPES, and 1% L-glutamine. When cells reached approximately 80–90% confluence, they were rinsed twice with PBS and incubated in serum-reduced medium (0.5% FBS) for 24 h to collect conditioned medium (CM). Supernatants were sequentially clarified (300× *g* for 5 min, followed by 2000× *g* for 10 min), passed through a 0.22 μm filter, and concentrated tenfold using Amicon Ultra centrifugal filters (10 kDa MWCO; Millipore, Merck KGaA, Darmstadt, Germany). CM aliquots were kept on ice throughout processing, protected from light, and stored at −20 °C. Each sample underwent only one freeze–thaw cycle before use.

Total protein in each CM batch was quantified using a BCA protein assay, and TIMP1 concentrations were measured by ELISA. For in vivo administration, all CM preparations were normalized to contain an identical total protein load (100 μg protein per mouse per injection, final volume 100 μL). Vector-CM served as the protein-matched control, and an additional PBS group was included. Where indicated as “blank medium,” heat-inactivated, FBS-free base medium was supplemented with BSA to match the total protein content of TIMP1-CM.

The per-dose TIMP1 payload in TIMP1-CM was 50 ± 8 ng (mean ± SEM, *n* = 3 batches), whereas vector-CM contained <1 ng TIMP1. Endotoxin levels were confirmed to be <0.1 EU/mL.

### 2.14. Flow Cytometry Analysis

Cells were trypsinized, washed three times with PBS, and resuspended in Cell Staining Buffer (BioLegend, San Diego, CA, USA). After cell counting, 1 × 10^6^ cells were sequentially stained with a viability dye 7-AAD (Yeasen Biotechnology, Shanghai, China), treated with Fc Blocking Reagent, and stained for surface markers including CD206 and CD163. Following surface staining, the cells were fixed, permeabilized, and incubated for 1 h at 4 °C with an intracellular antibody against iNOS. Cells were then washed three times with Cell Staining Buffer. Fluorescence data were acquired using a Sony ID7000™ flow cytometer (Sony Biotechnology Inc., San Jose, CA, USA) and analyzed with FlowJo™ software (version 10.9.0; BD Life Sciences, Ashland, OR, USA).

### 2.15. ELISA

Serum samples were allowed to clot at room temperature for 30 min and were then centrifuged at 2000× *g* for 15 min at 4 °C. TIMP1 concentrations were quantified using a TIMP1 ELISA Kit (Proteintech, KE00833, Wuhan, China) according to the manufacturer’s protocol. The required microplate strips were placed into the plate frame, and samples were loaded into the appropriate wells, including blank wells, standard wells, and test sample wells, all run in duplicate. Plates were sealed with adhesive film and incubated at 37 °C for 2 h. After incubation, wells were washed four times with 1× Wash Buffer by aspirating the liquid, followed by blotting dry against absorbent paper. Next, 100 µL of horseradish peroxidase (HRP)-conjugated detection antibody (1×) was added to each well and incubated at 37 °C for 40 min. Following another wash cycle, 100 µL of TMB substrate solution was added and developed at 37 °C in the dark for 15–20 min. The reaction was terminated by adding Stop Solution. Absorbance was measured at 450 nm with a correction wavelength of 630 nm using a microplate reader. A four-parameter logistic (4-PL) standard curve was generated using GraphPad Prism (version 7.0; GraphPad Software Inc., San Diego, CA, USA), plotting standard concentrations (*x*-axis) against mean OD values (*y*-axis). The interpolated concentration of each sample was calculated from the curve and multiplied by the dilution factor to determine the final concentration.

### 2.16. Murine Tumor Metastasis Models

An orthotopic colorectal cancer model was established by injecting 5 × 10^5^ MC38 or CT26 cells in 100 µL PBS into the cecal wall. On day 7, to induce liver metastasis, mice were anesthetized with isoflurane and received an intrasplenic injection of 5 × 10^5^ cells in 100 µL PBS, followed by splenectomy 10 min later. On day 28, mice were euthanized, and hepatic metastatic burden was quantified on H&E-stained sections as metastatic area/total liver area using ImageScope (version 12.4.6; Leica Biosystems Imaging Inc., Vista, CA, USA).

### 2.17. Drug and Immune Cell Depletion Treatments

Cilengitide was administered at 5 mg/kg, intraperitoneally (i.p.) in 200 μL, every 3 days from day −1 (one day before tumor implantation) until endpoint. For macrophage depletion, clodronate liposomes were given at 100 μL/mouse (≈5 mg/kg clodronate equivalent), i.p., daily on days 1–6, then every 3 days thereafter until endpoint. For neutrophil depletion, anti-Ly6G (1A8) antibodies were administered at 200 μg/mouse, i.p., daily on days 1–6, then every 3 days thereafter. For NK cell depletion, anti-NK1.1 (PK136) antibodies were administered at 200 μg/mouse, i.p., daily on days 1–6, then every 3 days thereafter. Depletion efficiency for Ly6G^+^ neutrophils and NK1.1^+^ NK cells was verified by flow cytometry on splenocytes collected at indicated time points.

### 2.18. Statistical Analysis

All data are presented as mean ± SEM. Statistical comparisons between groups were performed using Student’s *t*-test. Kaplan–Meier survival analysis was used to assess overall survival, with the log-rank test and univariate Cox regression applied to calculate hazard ratios (HRs) and 95% confidence intervals (CIs). Statistical analyses were conducted using SPSS version 17.0 (IBM, New York, NY, USA) or GraphPad Prism version 7.0 (GraphPad Software, USA). A *p*-value < 0.05 was considered statistically significant.

## 3. Results

### 3.1. TIMP1 Is Overexpressed in CRC and Correlates with Poor Prognosis

**Figure 1:**
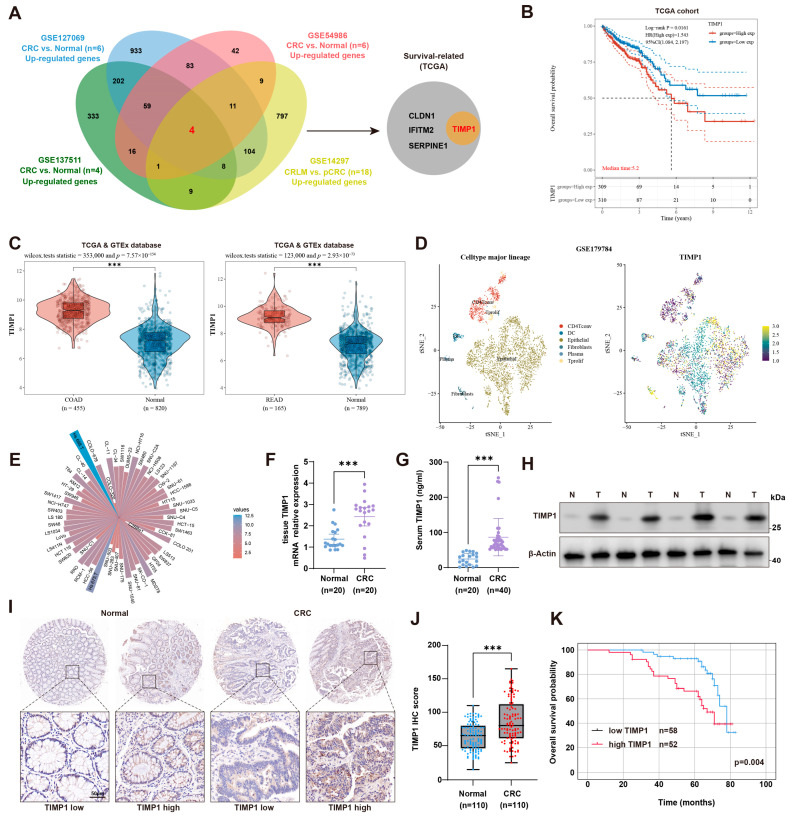
TIMP1 is overexpressed in CRC and correlates with poor prognosis. (**A**) Venn diagram showing the overlap of upregulated genes from four GEO datasets (GSE127069, GSE137511, GSE54986, and GSE14297), comparing normal tissues, primary CRC (pCRC), and colorectal liver metastases (CRLM). TIMP1, among other genes (CLDN1, IFITM2, SERPINE1), was identified as survival-related in the TCGA cohort. (**B**) Kaplan–Meier survival curves depicting overall survival in CRC patients from the TCGA cohort, stratified by TIMP1 expression levels (high vs. low). Median survival time is indicated. (**C**) Violin plots illustrating TIMP1 expression levels in normal tissues and CRC samples from the TCGA database. The left panel shows data for colon adenocarcinoma (COAD), and the right panel for rectal adenocarcinoma (READ). Statistical significance is indicated. (**D**) t-SNE plots derived from the GSE179784 dataset are shown, with cell type annotation presented in the left panel and TIMP1 expression patterns across different cell populations shown in the right panel. (**E**) A circular bar chart summarizes TIMP1 gene expression across a panel of colorectal cancer cell lines. Each bar corresponds to one cell line, and bar height reflects the relative expression level of TIMP1. (**F**,**G**) Quantification of TIMP1 mRNA expression in CRC tissues (*n* = 20) and TIMP1 protein levels in serum samples (*n* = 40). (**H**) Western blot showing TIMP1 protein levels in paired CRC tissues (T) and adjacent normal tissues (N). (**I**,**J**) Representative IHC images of TIMP1 expression in CRC and normal tissues (upper panels), with magnified views (lower panels) and corresponding IHC score comparison (*n* = 110). Scale bars: 50 µm. (**K**) Kaplan–Meier survival analysis of CRC patients stratified by TIMP1 IHC score. *p* value was calculated by Log-rank test. Data are presented as mean ± SEM. Statistical significance was assessed by unpaired two-tailed Student’s *t*-test (two groups) or one-way ANOVA with post hoc tests (≥3 groups) and by log-rank test for survival. *** *p* < 0.001.

To identify key genes associated with colorectal cancer (CRC) liver metastasis (CRLM), we performed an integrated transcriptomic analysis of four independent GEO datasets. Three datasets compared primary CRC tissues with matched normal mucosa (GSE127069, *n* = 6; GSE137511, *n* = 4; GSE54986, *n* = 6), and one dataset compared primary CRC with corresponding liver metastases (GSE14297, *n* = 18). Genes significantly up-regulated in CRC or CRLM in each cohort were then intersected to obtain the common candidate genes. A total of four overlapping genes were identified, among which TIMP1 was further confirmed to be associated with poor overall survival in the TCGA cohort (Figure 1A). Kaplan–Meier survival analysis revealed that patients with high TIMP1 expression had significantly worse overall survival than those with low expression (log-rank *p* < 0.001, HR = 2.171), with a median survival time of 5.2 years in the high-expression group (Figure 1B). To further validate the expression pattern of TIMP1 in CRC, its mRNA levels were compared between tumor and normal tissues using the TCGA and GTEx datasets. TIMP1 was significantly overexpressed in both colon adenocarcinoma (COAD) and rectal adenocarcinoma (READ) samples compared to adjacent normal tissues (Figure 1C). Single-cell RNA sequencing data from GSE179784 revealed that TIMP1 was predominantly expressed in epithelial cells (Figure 1D). Analysis of the CCLE database confirmed that TIMP1 was highly expressed across various CRC cell lines (Figure 1E). qPCR and ELISA assays confirmed significantly elevated TIMP1 mRNA expression in CRC tissues (*n* = 20) and increased circulating TIMP1 levels in the serum of CRC patients (*n* = 40) compared to normal controls (Figure 1F,G). Western blot analysis also validated upregulation of TIMP1 protein in paired CRC tissues (Figure 1H). Immunohistochemical analysis demonstrated markedly increased TIMP1 expression in CRC tissues, with heterogeneous staining patterns, whereas normal tissues showed weak or absent staining (Figure 1I). Immunohistochemical scoring further supported significantly higher TIMP1 expression in 110 CRC specimens compared with normal tissues (Figure 1J). Survival analysis stratified by IHC showed that patients with high TIMP1 protein expression had significantly worse overall survival than those with low expression (*p* = 0.004; Figure 1K). Clinicopathological correlation analysis revealed that high TIMP1 expression was significantly associated with lymph node metastasis (*p* = 0.043) and distant metastasis (*p* = 0.008), but showed no significant associations with gender, age, tumor size, tumor infiltration depth, or TNM stage (all *p* > 0.05; Additional file: Table S5). Univariate analysis identified TIMP1 expression (*p* = 0.004), lymph node metastasis (*p* = 0.019), and distant metastasis (*p* < 0.001) as significant factors influencing five-year overall survival. Multivariate Cox regression confirmed that high TIMP1 expression was an independent predictor of poor prognosis (HR = 4.644, *p* = 0.007), along with distant metastasis (HR = 5.424, *p* = 0.002; Additional file: Table S6). Collectively, through comprehensive multi-database bioinformatic analysis combined with multi-layer experimental validation, this study systematically demonstrated that TIMP1 is significantly upregulated in CRC. Moreover, its overexpression is strongly correlated with the immunosuppressive tumor microenvironment, metastatic behavior, and poor patient outcomes, suggesting that TIMP1 could serve as a promising prognostic biomarker for CRC and CRLM.

### 3.2. CRC Cells Can Secrete TIMP1 and Promote CRLM

**Figure 2:**
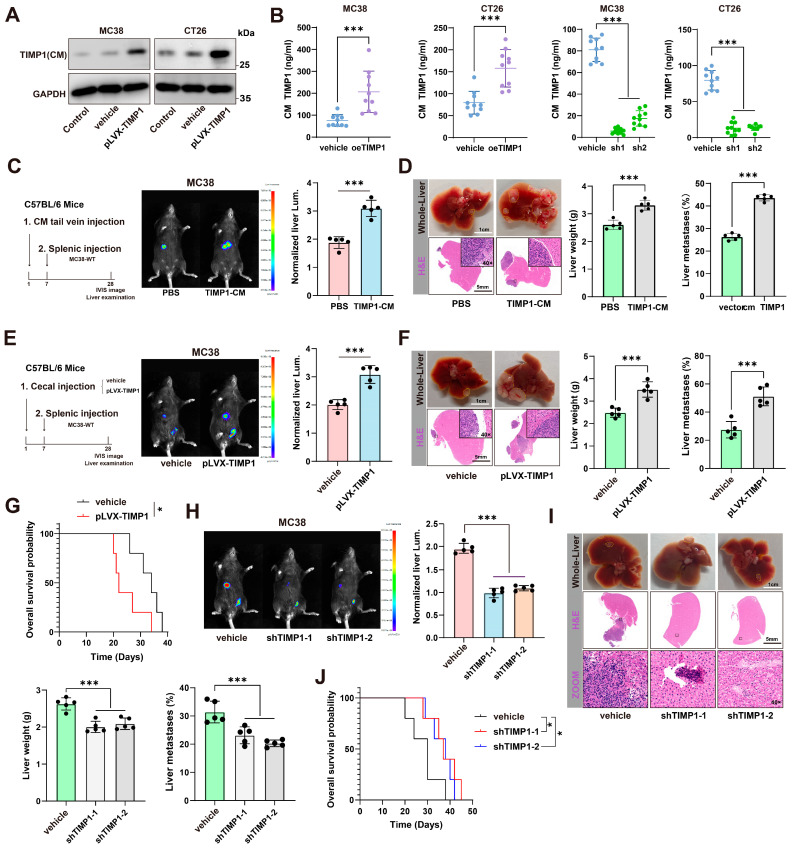
CRC-derived TIMP1 is secreted and primes the liver to promote metastasis. (**A**) Immunoblot of TIMP1 in conditioned media (CM) from MC38 and CT26 cells under control/vehicle or pLVX-TIMP1 overexpression. GAPDH, loading control of cell lysates. (**B**) ELISA quantification of TIMP1 in CM from MC38 and CT26 cells showing increased secretion upon oeTIMP1 and decreased secretion with two independent shRNA (sh1, sh2). (**C**) Liver-priming scheme in C57BL/6 mice: one intravenous dose of CM on day 1, day 7 intrasplenic injection of MC38-WT, and day 28 IVIS imaging and liver harvest. Representative IVIS images with quantification of normalized liver luminescence. Dose: protein-matched across groups; 100 μg total CM protein in 100 μL per mouse; TIMP1-CM 50 ± 8 ng/dose by ELISA; endotoxin < 0.1 EU/mL; vector-CM protein-matched; PBS vehicle. *n* = 5 mice/group. (**D**) Representative gross liver photographs and H&E staining (dashed outlines indicate metastatic areas) with liver-weight quantification for groups in (C). (**E**) Schematic of orthotopic priming: cecal implantation of MC38 vehicle or pLVX-TIMP1 cells, followed by intrasplenic injection of MC38-WT; representative IVIS liver images and quantification of normalized luminescence. *n* = 5 mice/group. (**F**) Corresponding gross/H&E liver images with quantification of liver weight and the percentage of liver metastases for groups in (E). (**G**) Kaplan–Meier overall-survival curves comparing vehicle and pLVX-TIMP1 groups. (**H**) Effects of TIMP1 knockdown (shTIMP1-1/-2) in MC38 cells on hepatic colonization: representative IVIS images and quantification of normalized liver luminescence. *n* = 5 mice/group. (**I**) Gross and H&E liver images with liver-weight and metastatic-percentage quantification for the knockdown cohorts in (H). (**J**) Kaplan–Meier overall survival analysis for vehicle and shTIMP1 groups. Data are presented as mean ± SEM. Statistical significance was assessed by unpaired two-tailed Student’s *t*-test (two groups) or one-way ANOVA with post hoc tests (≥3 groups) and by log-rank test for survival. * *p* < 0.05, *** *p* < 0.001.

TIMP1, a soluble protein secreted into the extracellular environment, is widely present in most tissues and body fluids [17,18]. However, its role as a secreted protein in modulating the tumor microenvironment has not been fully validated in animal models to date. To address this, we established stable TIMP1 knockdown and overexpression cell lines in both murine CRC cell lines (MC38 and CT26) and human CRC cell lines (HCT116 and SW480). qPCR analysis confirmed efficient reduction and elevation of TIMP1 mRNA levels following shRNA-mediated knockdown or lentiviral overexpression, respectively (Supplementary Figure S2A,B). At the protein level, Western blot of conditioned media further demonstrated that endogenous TIMP1 secretion was markedly decreased by two independent shTIMP1 constructs and increased by pLVX-TIMP1 in both murine and human CRC cells (Figure 2A and Supplementary Figure S2C,D). Consistently, ELISA measurements of conditioned media showed that TIMP1 secretion rose with overexpression and fell with shRNA-mediated knockdown in MC38 and CT26 cells (Figure 2B).

For functional assays in C57BL/6 mice, we infused protein-normalized, BCA-matched conditioned medium through the tail vein on day 1. Each dose contained 100 μg total protein in 100 μL per mouse, and TIMP1-CM delivered 50 ± 8 ng TIMP1 per dose by ELISA with endotoxin below 0.1 EU/mL. Protein-matched vector-CM and PBS served as controls. On day 7, wild-type MC38 cells were injected intrasplenically. On day 28, bioluminescence imaging quantified hepatic colonization, and livers were harvested for H&E to verify and measure metastatic burden. Mice preconditioned with TIMP1-CM showed higher liver bioluminescence, increased liver weight, and a greater percentage of metastatic involvement compared with vector-CM or PBS, indicating that CRC-derived TIMP1 preconditions the liver to support metastatic outgrowth (Figure 2C,D). We next asked whether sustained tumor-intrinsic TIMP1 production promotes metastasis from an orthotopic site. Using a cecal implantation model with vehicle- or pLVX-TIMP1-transduced MC38 cells, followed by splenic injection of MC38-WT cells, enforced TIMP1 expression markedly increased liver bioluminescence, liver weight, and the percentage of metastatic involvement, and significantly shortened overall survival (Figure 2E–G). Conversely, knockdown of TIMP1 using two independent shRNA constructs (shTIMP1-1 and shTIMP1-2) significantly reduced liver metastatic burden and tumor-associated liver enlargement (Figure 2H,I). Survival analysis demonstrated that TIMP1 knockdown significantly prolonged the overall survival of tumor-bearing mice compared to vehicle-treated controls (Figure 2J), indicating that TIMP1 plays a critical role in promoting CRLM and contributes to poor prognosis.

### 3.3. TIMP1 Promotes CRLM in a Macrophage-Dependent Manner

**Figure 3:**
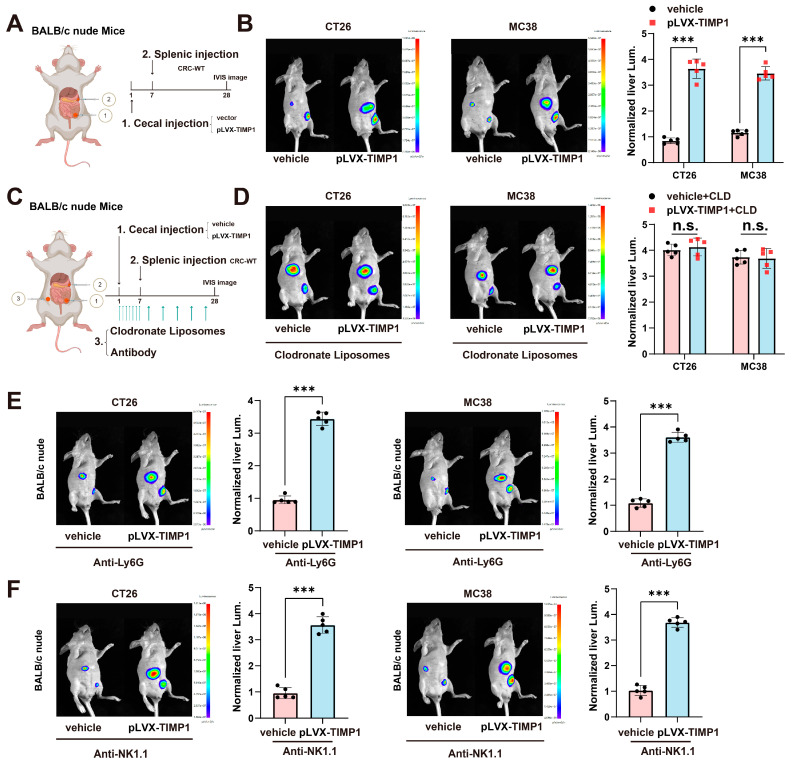
TIMP1 promotes CRLM in a macrophage-dependent manner. (**A**) Schematic diagram of the orthotopic CRC liver metastasis model. BALB/c nude mice were implanted with CT26 or MC38 cells transduced with pLVX-TIMP1 or vector control into the cecal wall, followed seven days later by intrasplenic injection of wild-type CRC cells to mimic hematogenous dissemination. (**B**) Representative in vivo bioluminescence (IVIS) images of the liver region and corresponding quantification of normalized liver luminescence in control versus TIMP1-overexpressing groups. *n* = 5 mice/group. (**C**) Experimental strategy for macrophage depletion: clodronate liposomes were administered i.p. starting on the day of cecal implantation (day 1) at 100 μL/mouse (~5 mg/kg clodronate equivalent), daily on days 1–6, then every 3 days thereafter until endpoint; PBS-loaded liposomes served as control. *n* = 5 mice/group. (**D**) Representative IVIS images and quantification of liver luminescence in control and TIMP1-overexpressing groups with or without clodronate liposome treatment. *n* = 5 mice/group. (**E**) Representative IVIS images and quantification of liver luminescence after neutrophil depletion with anti-Ly6G (clone 1A8): 200 μg/mouse i.p., starting on day 1, daily on days 1–6, then every 3 days thereafter until endpoint; depletion verified by flow cytometry. *n* = 5 mice/group. (**F**) Representative IVIS images and quantification of liver luminescence after NK-cell depletion with anti-NK1.1 (clone PK136): 200 μg/mouse i.p., starting on day 1, daily on days 1–6, then every 3 days thereafter until endpoint; depletion verified by flow cytometry. *n* = 5 mice/group. Data are shown as mean ± SEM, with each dot representing one animal. Statistical analysis was performed using one-way ANOVA followed by post hoc testing. *** *p* < 0.001; n.s., not significant. All IVIS images were acquired using identical exposure and filter settings, and quantification was performed on fixed-size regions of interest (ROIs) over the liver, background-subtracted and normalized for comparison across groups.

To elucidate the immune cell–dependent mechanisms underlying the pro-metastatic function of TIMP1, we employed immunodeficient mouse models in combination with specific immune cell depletion strategies. TIMP1 has been implicated in multiple aspects of tumor progression within the tumor microenvironment, although the specific immune cell populations responsible for its tumor-promoting effects remain uncleard [19]. To assess whether TIMP1-driven liver metastasis depends on T cells, we implanted CT26 or MC38 colorectal cancer cells stably overexpressing TIMP1 into BALB/c nude mice, which lack functional T cells (Figure 3A). Compared with vector controls, TIMP1 overexpression significantly increased liver metastatic burden in these mice, as detected by in vivo bioluminescence imaging (Figure 3B). These findings indicate that the pro-metastatic effects of TIMP1 are independent of T cells, thereby suggesting that other immune cell populations may be responsible for mediating its tumor-promoting activity.

To explore the role of macrophages in mediating TIMP1-driven metastasis, we depleted macrophages using clodronate liposomes (CLD) as schematized in (Figure 3C). Mice received clodronate liposomes intraperitoneally starting on the day of cecal implantation (day 1) at 100 μL per mouse (5 mg/kg clodronate equivalent), daily on days 1–6, then every 3 days thereafter until the endpoint. Notably, in CLD-treated mice, TIMP1-induced enhancement of liver metastasis was completely abolished. There were no significant differences in liver fluorescence intensity between vehicle and pLVX-TIMP1 groups in either CT26 or MC38 models (Figure 3D), demonstrating that macrophages are indispensable for TIMP1-mediated metastatic promotion.

To further test whether TIMP1-conditioned macrophages are sufficient to promote liver colonization, we performed an adoptive transfer experiment. Murine macrophages were pretreated in vitro with PBS or recombinant TIMP1 and then co-injected with CT26 cells into BALB/c nude mice via intrasplenic injection. Compared with mice receiving PBS-treated macrophages, mice co-injected with TIMP1-treated macrophages displayed increased liver weight and a higher percentage of liver surface occupied by metastases (Supplementary Figure S3A–C). These data provide additional functional evidence that TIMP1-educated macrophages directly facilitate colorectal cancer liver colonization in vivo.

To determine whether other innate immune populations such as neutrophils or natural killer (NK) cells are involved in TIMP1-driven metastasis, we performed antibody-mediated depletion experiments. Neutrophils were depleted using anti-Ly6G monoclonal antibody (clone 1A8; 200 µg/mouse, i.p.) once daily on days 1–6 and then every 3 days thereafter, on the same schedule as clodronate liposomes.

Interestingly, neither neutrophil nor NK cell depletion had any discernible impact on TIMP1-induced liver metastasis. As shown in (Figure 3E), mice receiving anti-Ly6G still exhibited significantly increased liver fluorescence intensity upon TIMP1 overexpression in both tumor models, suggesting that neutrophils are not essential mediators of TIMP1’s metastatic function. Similarly, depletion of NK cells using anti-NK1.1 (Figure 3F) failed to suppress TIMP1-mediated metastasis, further excluding a role for NK cells in this context.

Collectively, these results indicate that macrophages are the critical immune cell type responsible for transducing TIMP1’s pro-metastatic effects in colorectal cancer.

### 3.4. TIMP1 Stimulation Reprograms Liver Macrophage Gene Expression

**Figure 4:**
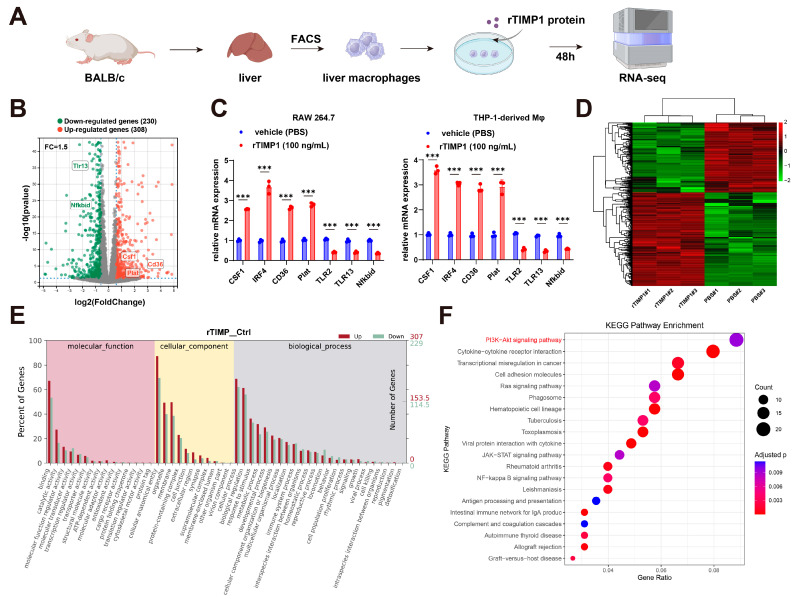
TIMP1 stimulation reprograms liver macrophage gene expression. (**A**) Schematic of the experimental design: Liver macrophages isolated from BALB/c mice were treated with recombinant TIMP1 (rTIMP1, 100 ng/mL) for 48 h, followed by RNA sequencing. (**B**) Volcano plot showing differentially expressed genes between rTIMP1-treated and control macrophages (fold change > 1.5, adjusted *p* < 0.05). Red: upregulated genes (*n* = 308); green: downregulated genes (*n* = 230); gray: non-significant. Selected genes are labeled. (**C**) qPCR validation of representative genes in RAW264.7 and THP-1-derived macrophages treated with rTIMP1 or vehicle (PBS). Data represent mean ± SEM (*n* = 3). *** *p* < 0.001, unpaired two-tailed Student’s *t*-test. (**D**) Heatmap showing hierarchical clustering of differentially expressed genes in rTIMP1-treated and control groups. (**E**) Gene Ontology (GO) enrichment analysis of upregulated (red) and downregulated (green) genes in the categories of molecular function, cellular component, and biological process. (**F**) KEGG pathway enrichment analysis of differentially expressed genes. Size of the bubbles represents gene count; color indicates adjusted *p*-value. Key pathways including PI3K-Akt, cytokine–cytokine receptor interaction, and JAK-STAT signaling are enriched in the rTIMP1-treated group.

To examine the immunomodulatory effects of TIMP1, we prepared single-cell suspensions from BALB/c livers and isolated hepatic macrophages by FACS using a stepwise gating strategy: singlets (FSC-H vs. FSC-A), forward/side scatter to exclude debris (FSC-A vs. SSC-A), viable cells (7-AAD−), leukocytes (CD45+), and macrophages defined as F4/80+CD11b+ (Supplementary Figure S4A). The indicated fractions were sorted, cultured with or without recombinant TIMP1 for 48 h, and subjected to RNA-seq (Figure 4A). Differential gene expression analysis identified 308 upregulated and 230 downregulated genes in the rTIMP1-treated group (fold change > 1.5, adjusted *p* < 0.05) (Figure 4B). Notably, M2-associated genes such as CSF1, IRF4, and CD36 were upregulated, whereas several pro-inflammatory genes including Tlr3, Nfkbid, and Tlr13 were downregulated. Validation of key targets by qPCR in both RAW264.7 and THP-1-derived macrophages confirmed that rTIMP1 treatment significantly increased mRNA expression of CSF1, IRF4, CD36, and Plat, along with reduced levels of Tlr3, Tlr13, and Nfkbid, supporting a shift toward an anti-inflammatory macrophage phenotype (Figure 4C).

Unsupervised hierarchical clustering of RNA-seq data further demonstrated distinct transcriptional profiles between rTIMP1-treated and vehicle groups, with clear separation of differentially expressed genes (Figure 4D). Gene Ontology (GO) analysis revealed that rTIMP1-regulated genes were enriched in terms associated with immune regulation, membrane components, and cytokine activity (Figure 4E). In parallel, KEGG pathway analysis identified significant enrichment of the PI3K–Akt signaling pathway, cytokine–cytokine receptor interaction, and JAK–STAT signaling, suggesting that TIMP1 influences multiple immune-related signaling pathways (Figure 4F).

### 3.5. TIMP1 Drives Macrophage M2 Polarization and Pre-Metastatic Niche Formation

**Figure 5:**
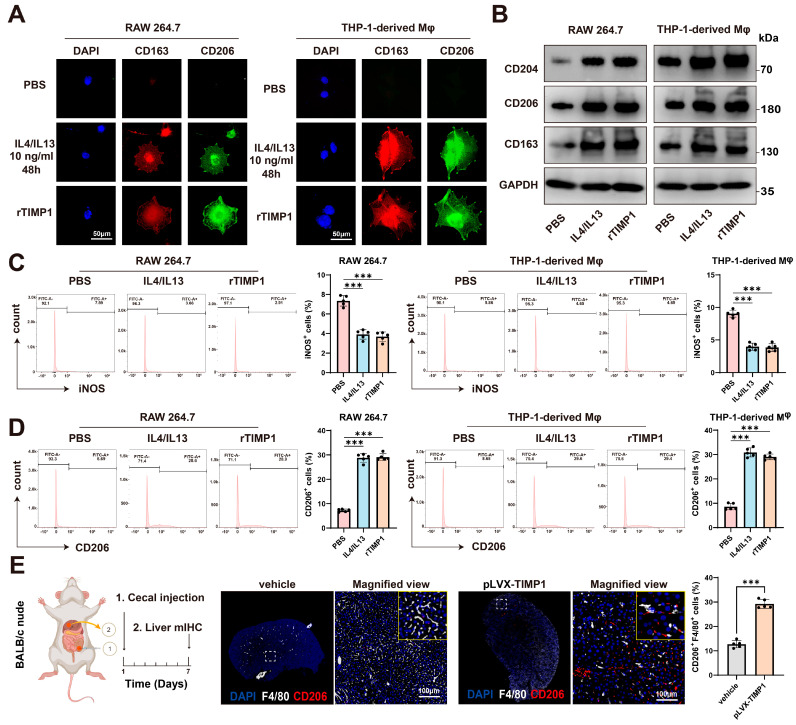
TIMP1 drives macrophage M2 polarization and pre-metastatic niche formation. (**A**) Immunofluorescence staining of RAW264.7 and THP-1-derived macrophages treated with PBS, IL4/IL13 (10 ng/mL, 48 h), or recombinant TIMP1 (rTIMP1). Cells were stained for CD163 (red) and CD206 (green); nuclei were counterstained with DAPI (blue). Scale bars: 50 µm. (**B**) Western blot analysis of M2 markers (CD204, CD206, CD163) in RAW264.7 and THP-1-derived macrophages following treatment as indicated. GAPDH served as a loading control. (**C**) Flow cytometric analysis and quantification of iNOS-positive macrophages in RAW264.7 and THP-1-derived macrophages after PBS, IL4/IL13, or rTIMP1 treatment. (**D**) Flow cytometric analysis and quantification of CD206-positive macrophages in the same groups. (**E**) Schematic of the cecal orthotopic injection model in BALB/c nude mice. Multiplex immunohistochemistry of liver tissues showing F4/80 (white) and CD206 (red) co-expression. Quantification indicates increased CD206^+^F4/80^+^ macrophages in TIMP1-overexpressing group compared with control. Scale bars: 100 µm. Data are presented as mean ± SEM, *** *p* < 0.001.

To examine whether TIMP1 affects macrophage polarization, we analyzed the expression of M2-associated markers in murine RAW264.7 macrophages and human THP-1–derived macrophages. Immunofluorescence staining showed that treatment with IL4/IL13 or recombinant TIMP1 (rTIMP1) increased the expression of CD163 and CD206 compared with PBS-treated controls, consistent with an M2-like polarization phenotype (Figure 5A). Consistently, Western blot analysis confirmed that rTIMP1 promoted upregulation of M2-associated markers (CD204, CD206, and CD163) in both cell types, comparable to IL4/IL13 stimulation (Figure 5B).

Next, flow cytometric analysis demonstrated that rTIMP1 treatment significantly reduced the proportion of iNOS-positive macrophages (a representative M1 marker) (Figure 5C), while increasing the frequency of CD206-positive macrophages (Figure 5D), further supporting its role in driving M2 polarization.

Given that the hepatic immune microenvironment undergoes reprogramming prior to the arrival of tumor cells to establish a pre-metastatic niche, we further investigated the role of TIMP1 in this process. TIMP1-overexpressing CT26 cells were orthotopically injected into the cecum of nude mice, and livers were collected for multiplex immunohistochemistry (mIHC) seven days later. The results revealed a significantly higher proportion of F4/80 and CD206 double-positive M2-like macrophages in the TIMP1-overexpression group compared with controls (*p* < 0.05), suggesting that CRC-derived TIMP1 promotes the accumulation of M2-like macrophages within the liver immune microenvironment to establish a pre-metastatic niche (Figure 5E). In summary, CRC-derived TIMP1 can induce macrophage M2 polarization, facilitating immune evasion and remodeling the liver microenvironment to establish a pre-metastatic niche, thereby enhancing the survival and colonization capacity of colorectal cancer cells in the liver.

### 3.6. TIMP1 Interacts with CD63 and β1-Integrin to Activate AKT/mTOR Signaling and Promote M2 Macrophage Polarization

**Figure 6:**
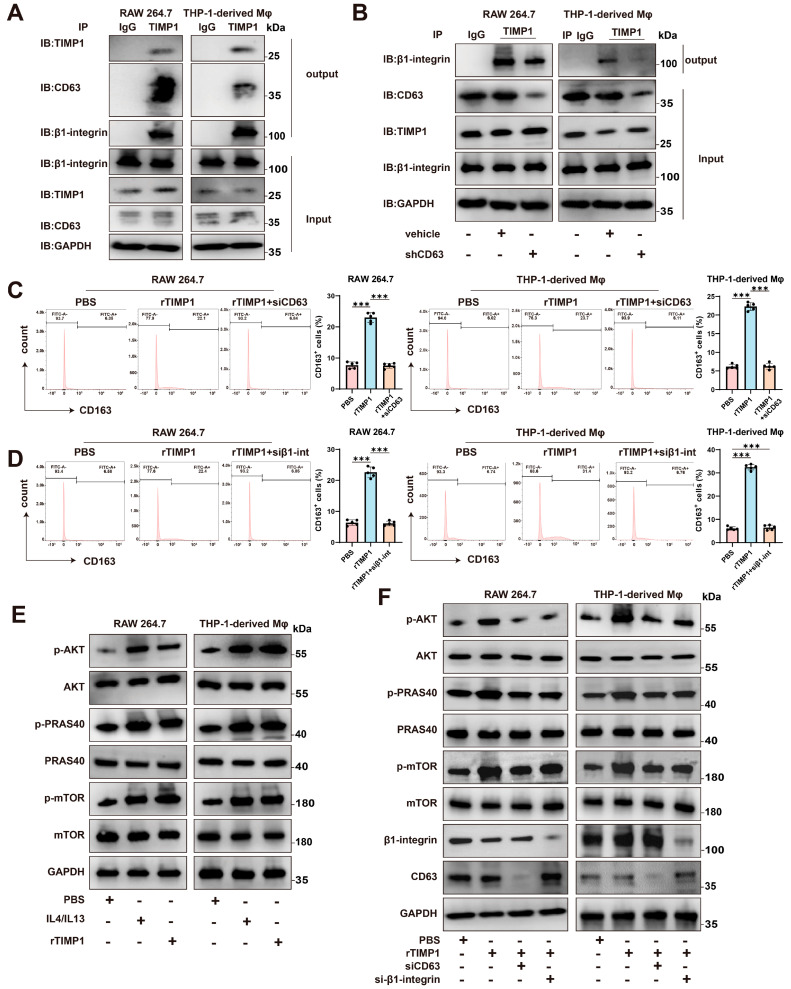
TIMP1 binds to CD63/β1-integrin on macrophages and activates the AKT/mTOR pathway. (**A**) Co-immunoprecipitation (Co-IP) showing the interaction between TIMP1 and CD63/β1-integrin in RAW264.7 and THP-1-derived macrophages. Cell lysates were immunoprecipitated with anti-TIMP1 or control IgG and immunoblotted for CD63, β1-integrin, and TIMP1. (**B**) Reverse Co-IP using anti-β1-integrin confirms the formation of a TIMP1-CD63-β1-integrin complex. (**C**,**D**) Flow cytometric analysis of CD163^+^ macrophages after treatment with PBS, rTIMP1, rTIMP1 + siCD63, or rTIMP1 + si-β1-integrin. Quantification of CD163^+^ cell percentages is shown (right). (**E**) Western blot analysis of p-AKT, p-PRAS40, and p-mTOR in macrophages treated with PBS, rTIMP1, or rTIMP1 + IL4/IL13. (**F**) Knockdown of CD63 or β1-integrin inhibits TIMP1-induced activation of the AKT/mTOR signaling pathway. GAPDH was used as a loading control. Data are presented as mean ± SEM from at least three independent experiments. Statistical significance: *** *p* < 0.001.

To investigate the molecular mechanism by which TIMP1 modulates macrophage polarization, we first examined whether TIMP1 physically interacts with known surface receptors in macrophages. Co-immunoprecipitation (Co-IP) assays using RAW264.7 and THP-1-derived macrophages revealed that TIMP1 forms complexes with both CD63 and β1-integrin (Figure 6A). Immunoprecipitation of endogenous TIMP1 resulted in co-precipitation of β1-integrin in both cell types, whereas no corresponding signal was observed in IgG control samples, supporting the specificity of this interaction. In addition, knockdown of CD63 reduced the association between TIMP1 and β1-integrin, indicating that CD63 contributes to the formation of this complex (Figure 6B).

We next assessed the functional relevance of CD63 and β1-integrin in TIMP1-induced macrophage polarization. Flow cytometric analysis showed that treatment with recombinant TIMP1 increased the proportion of CD163⁺ M2-like macrophages relative to PBS-treated controls. In contrast, silencing of CD63 or β1-integrin using specific siRNAs attenuated the rTIMP1-induced increase in CD163⁺ cells, indicating that both molecules are required for efficient induction of the M2 phenotype (Figure 6C,D).

Given the established role of the AKT/mTOR pathway in macrophage polarization and immunometabolic regulation, we further examined whether this pathway is involved in TIMP1-mediated signaling. Western blot analysis demonstrated that rTIMP1 treatment increased phosphorylation of AKT, PRAS40, and mTOR in both RAW264.7 and THP-1–derived macrophages (Figure 6E). This effect was further enhanced in the presence of IL4 and IL13, classical M2-polarizing cytokines, suggesting a potential synergistic mechanism between TIMP1 and Th2-type cytokines. Importantly, silencing CD63 or β1-integrin abolished TIMP1-induced phosphorylation of AKT, PRAS40, and mTOR (Figure 6F), further supporting that TIMP1 exerts its immunomodulatory function through CD63/β1-integrin-mediated activation of the AKT/mTOR axis.

### 3.7. Cilengitide Blocks TIMP1-Induced M2 Polarization and Reduces Liver Metastasis In Vivo

**Figure 7:**
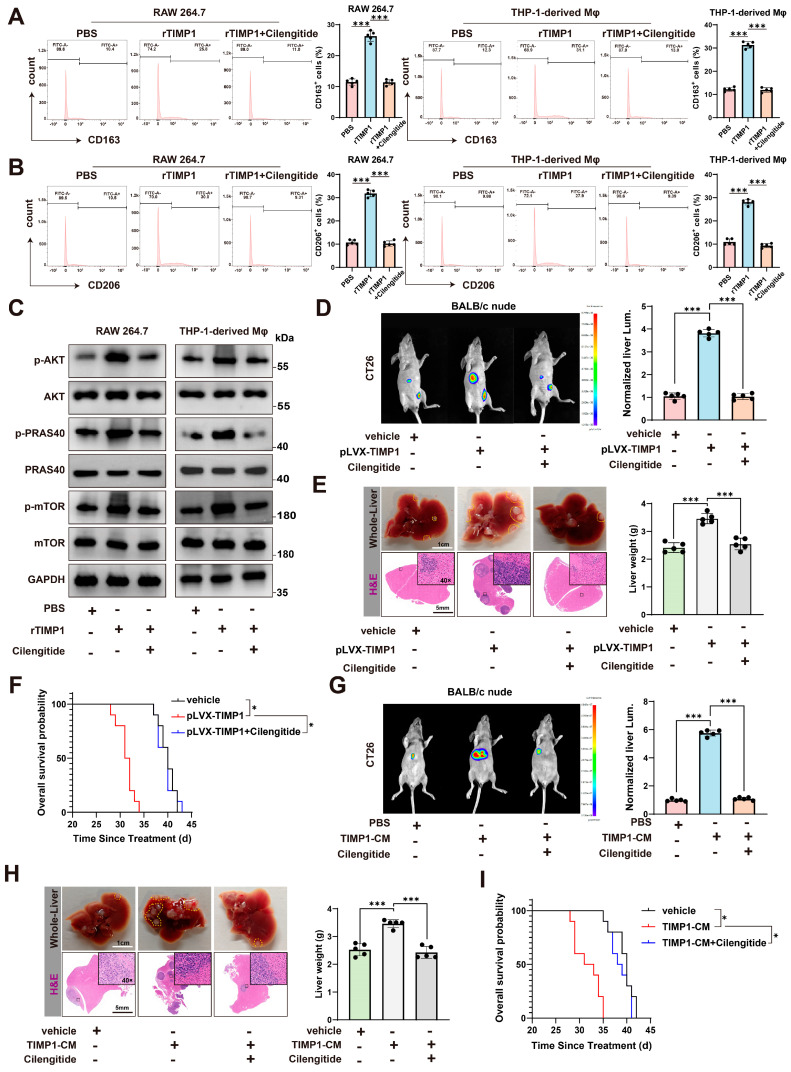
Cilengitide blocks the TIMP1/CD63/β1-integrin/mTOR axis and inhibits M2-macrophage-mediated CRLM. (**A**) Flow cytometry of CD163^+^ macrophages in RAW264.7 and THP-1–derived macrophages treated with PBS, recombinant TIMP1 (rTIMP1 100 ng/mL), or rTIMP1 plus Cilengitide 5 μM for 24 h; quantification on the right. (**B**) CD206^+^ macrophages measured as in (A) under the same treatments and timing. (**C**) Immunoblot of p-/total AKT, PRAS40, and mTOR in RAW264.7 and THP-1–derived macrophages after 24 h of PBS, rTIMP1 100 ng/mL, or rTIMP1 + Cilengitide 5 μM; GAPDH, loading control. (**D**) Orthotopic model in BALB/c nude mice with CT26: representative IVIS images and quantification of normalized liver luminescence showing enhanced metastatic burden with pLVX-TIMP1 and its suppression by Cilengitide. Cilengitide 5 mg/kg, i.p., in 200 μL every 3 days from day −1 to endpoint. (**E**) Corresponding whole-liver photos and H&E images (dashed lines, metastatic areas) with liver-weight quantification for the groups in (D). (**F**) Kaplan–Meier overall survival for vehicle, pLVX-TIMP1, and pLVX-TIMP1 + Cilengitide. (**G**) Validation using TIMP1-conditioned medium (TIMP1-CM) with CT26: representative liver IVIS images and quantification for PBS, TIMP1-CM, and TIMP1-CM + Cilengitide. One intravenous dose of CM on day 1, protein-matched across groups, 100 μg total protein in 100 μL per mouse; TIMP1 50 ± 8 ng per dose by ELISA; PBS as vehicle. Cilengitide 5 mg/kg, i.p., every 3 days starting day −1. (**H**) Gross liver and H&E images with liver-weight quantification for the groups in (G). (**I**) Kaplan–Meier overall survival for PBS, TIMP1-CM, and TIMP1-CM + Cilengitide. Data represent mean ± SEM. Statistical analysis: one-way ANOVA or log-rank test; * *p* < 0.05, *** *p* < 0.001.

To determine whether targeting the integrin signaling axis could neutralize the pro-tumoral effects of TIMP1, we treated macrophages with cilengitide, a cyclic RGD peptide inhibitor that selectively blocks αvβ3 and αvβ5 integrins, and partially interferes with β1-integrin-related signaling [20,21]. Flow cytometry analysis showed that treatment with cilengitide significantly reduced the percentage of CD163^+^ and CD206^+^ cells among rTIMP1-treated macrophages, in both RAW264.7 and THP-1-derived models (Figure 7A,B), indicating inhibition of M2 marker expression, and suggesting disruption of the TIMP1-induced polarization program. In line with the β1-integrin dependence suggested by our genetic data, a β1-integrin-blocking antibody (AIIB2) similarly attenuated rTIMP1-induced CD163^+^ and CD206^+^ macrophage polarization in both models (Supplementary Figure S5A,B).

Consistent with this, cilengitide effectively abolished rTIMP1-induced activation of the AKT/mTOR signaling pathway, as shown by decreased phosphorylation of AKT, PRAS40, and mTOR (Figure 7C). Moreover, direct pharmacologic inhibition downstream of integrins using the AKT inhibitor MK2206 (MedChemExpress, Monmouth Junction, NJ, USA) or the mTOR inhibitor rapamycin (Sigma-Aldrich, St. Louis, MO, USA) reduced the proportion of CD163^+^ and CD206^+^ cells in response to rTIMP1 (Supplementary Figure S5C,D), providing additional mechanistic support that AKT/mTOR signaling is required for TIMP1-driven M2 polarization.

We next evaluated the in vivo consequences of TIMP1 overexpression and cilengitide treatment using a colorectal cancer liver metastasis model. In BALB/c nude mice inoculated with CT26 cells overexpressing TIMP1 (pLVX-TIMP1), bioluminescence imaging revealed a marked increase in liver metastatic burden compared to vehicle controls. However, treatment with cilengitide significantly reduced hepatic tumor signal intensity (Figure 7D). Gross morphological inspection and H&E staining of liver tissues confirmed a substantial reduction in metastatic nodules and liver weight in the cilengitide-treated group (Figure 7E). Moreover, mice in the pLVX-TIMP1 group exhibited significantly shortened survival, whereas cilengitide treatment prolonged overall survival, restoring it close to control levels (Figure 7F). Consistent with a role for β1-integrin signaling in this process, AIIB2 treatment in the CT26-pLVX-TIMP1 model partially reversed the increase in liver weight and metastatic burden induced by TIMP1 overexpression (Supplementary Figure S5E).

To validate these findings in an independent model, we used MC38 colorectal cancer cells and TIMP1-conditioned medium (TIMP1-CM) to mimic TIMP1-rich tumor environments. Again, cilengitide treatment markedly suppressed liver metastasis, as evidenced by reduced liver flux signals (Figure 7G), liver weights, and histological metastatic burden (Figure 7H). Survival analysis also demonstrated that mice receiving TIMP1-CM had significantly shorter survival, which was rescued by cilengitide treatment (Figure 7I).

Taken together, these results highlight that TIMP1 promotes macrophage M2 polarization and colorectal cancer liver metastasis via integrin-mediated signaling, and that cilengitide represents a promising therapeutic strategy to disrupt this axis.

## 4. Discussion

In this study, we identified tissue inhibitor of metalloproteinases-1 (TIMP1) as a key mediator in colorectal cancer (CRC) liver metastasis (CRLM), acting through a macrophage-dependent mechanism. We demonstrated that TIMP1 is significantly overexpressed in CRC tissues and correlates with poor patient prognosis. Importantly, TIMP1 was shown to modulate the tumor immune microenvironment by inducing M2 macrophage polarization via CD63/β1-integrin-mediated activation of the AKT/mTOR signaling pathway, thereby promoting hepatic pre-metastatic niche (PMN) formation and metastatic colonization. Consistent with this mechanism, pharmacological blockade with cilengitide reduced TIMP1-driven signaling activity and attenuated metastatic progression, supporting the potential of targeting macrophage plasticity in CRLM.

The formation of the PMN represents a key early event in metastatic dissemination and is shaped by multiple tumor-derived soluble factors, including extracellular matrix (ECM) remodeling proteins, chemokines, and inflammatory mediators [7,22]. Previous studies have shown that TIMP1 can enhance SDF-1 expression during PMN development, thereby promoting neutrophil recruitment to the liver and increasing susceptibility to metastatic seeding [23,24]. In addition, TIMP1 has been implicated in ECM remodeling, inflammatory activation, epithelial–mesenchymal transition (EMT), and angiogenesis, collectively contributing to increased tumor cell invasiveness and adaptability [25,26]. Our data further extend these observations by demonstrating elevated TIMP1 secretion in primary CRC tissues and increased levels at liver metastatic sites, suggesting that TIMP1 may precondition the hepatic microenvironment through paracrine mechanisms. Moreover, TIMP1-driven macrophage reprogramming toward an M2-like, immunosuppressive phenotype appears to support the establishment of a pro-metastatic niche.

Consistent with prior reports indicating the oncogenic role of TIMP1 in various cancers [27,28,29], our bioinformatic and clinical analyses confirmed that TIMP1 is overexpressed in CRC tissues and patient sera, and its elevated expression is independently associated with poor overall survival. These findings suggest that TIMP1 may serve not only as a diagnostic and prognostic biomarker but also as a candidate therapeutic target, particularly for patients at high risk for liver metastasis.

A notable strength of the present study lies in uncovering the functional role of TIMP1 as a secreted factor that modulates the immune landscape of the liver microenvironment [30,31]. Using both in vitro and in vivo models, we demonstrate that CRC-derived TIMP1 promotes liver metastasis through macrophage reprogramming. Importantly, this pro-metastatic effect was preserved in immunodeficient BALB/c nude mice lacking functional T cells, but was substantially reduced following macrophage depletion, indicating a central role for macrophages in TIMP1-mediated metastatic progression. These observations are consistent with accumulating evidence that tumor-associated macrophages (TAMs), particularly those exhibiting an M2-like phenotype, contribute to immunosuppression, angiogenesis, and metastatic dissemination [32,33,34].

At the mechanistic level, our data provide evidence that TIMP1 induces M2 polarization of macrophages through direct interaction with CD63 and β1-integrin, leading to activation of the AKT/PRAS40/mTOR signaling axis. This interaction was supported by co-immunoprecipitation experiments and functional loss-of-function analyses, which showed that disruption of CD63 or β1-integrin impaired downstream signaling and reduced M2 marker expression. These results are consistent with previous reports identifying CD63 as a functional receptor for TIMP1 and a mediator of intracellular signaling [14,15]. Notably, our study expands on this knowledge by identifying the integrin-dependent AKT/mTOR pathway as a downstream effector in macrophages, a cell type that has not been extensively investigated in this context.

Through transcriptomic profiling and cytokine analysis, we identified a suite of TIMP1-induced immunomodulatory genes, including CSF1, IRF4, and CD36, which further reinforced the M2 phenotype and established a feedforward loop that amplifies immune suppression within the PMN. Our immunofluorescence staining and flow cytometry results confirmed the enrichment of CD206+F4/80+ M2-like macrophages in the livers of mice bearing TIMP1-overexpressing tumors even before visible metastases occurred. Together, these findings highlight the role of TIMP1 in shaping the pre-metastatic liver microenvironment and enhancing metastatic fitness.

Of translational importance, we demonstrate that Cilengitide, a well-characterized RGD-mimetic integrin antagonist, effectively inhibits TIMP1-induced AKT/mTOR activation and M2 polarization. Clinically, cilengitide has been evaluated in multiple phase I–III trials in cancer, most prominently in glioblastoma and also in other solid tumours, where it was generally well tolerated and showed evidence of target engagement but led to only modest, context-dependent improvements in outcome when added to standard chemoradiotherapy or chemotherapy [35,36]. These mixed results suggest that integrin blockade is unlikely to be broadly effective as an unselected monotherapy and that its benefit may depend on specific biological niches [37]. In our models, Cilengitide treatment suppressed liver metastasis in vivo, supporting the concept that integrin inhibition may be most useful in biomarker-defined settings such as TIMP1-high, macrophage-driven CRLM, rather than in unstratified patient populations. Future studies combining integrin antagonists with immunotherapy or chemotherapy, and selecting patients on the basis of TIMP1–integrin pathway activation, will be important to explore. Importantly, the limited efficacy observed in unselected clinical trials highlights that further preclinical studies are needed to define the biological contexts most likely to respond. In this regard, biomarker-guided patient stratification—such as TIMP1 expres-sion profiling and identification of macrophage-dependent CRLM with evidence of CD63/β1-integrin–AKT/mTOR activation—may be critical to translate integrin blockade into meaningful clinical benefit.

Several limitations of this study should be considered. Although the mouse models used here reproduce key features of human CRC liver metastasis, differences in macrophage biology between species may influence the extent to which these findings can be translated to human disease. In addition, immune evasion mechanisms in human tumors are likely to be more complex than those captured by current murine systems, which may partially explain the limited clinical efficacy observed for integrin antagonists in earlier trials. It therefore remains unclear whether these discrepancies primarily reflect fundamental differences between murine and human tumor immunology or instead highlight the need for improved preclinical models, such as those that more closely mimic the human liver metastatic niche. Furthermore, while our study focused on CD63 and β1-integrin as key mediators of TIMP1 signaling, it is possible that additional co-receptors or regulatory molecules also contribute to TIMP1-dependent effects and warrant further investigation. Finally, the precise temporal window during which TIMP1 exerts its PMN-modulating activity has not yet been fully defined.

In conclusion, our study provides compelling evidence that CRC-secreted TIMP1 reprograms liver macrophages toward an immunosuppressive M2 phenotype via CD63/β1-integrin–mediated AKT/mTOR signaling, thereby promoting PMN formation and liver metastasis. These findings not only advance our understanding of the immunoregulatory roles of TIMP1 but also offer a rationale for targeting TIMP1-driven macrophage polarization as a novel therapeutic strategy in CRC. Future efforts should explore combination regimens incorporating TIMP1 or integrin inhibitors to improve outcomes in metastatic CRC.

## 5. Conclusions

This study demonstrates that CRC-secreted TIMP1 drives liver metastasis by reprogramming macrophages toward an M2 phenotype through CD63/β1-integrin–AKT/mTOR signaling. TIMP1 expression correlates with poor prognosis and promotes pre-metastatic niche formation. Of note, integrin blockade with cilengitide abrogated TIMP1-induced macrophage polarization and metastasis. These findings highlight TIMP1 as a prognostic biomarker and a potential therapeutic target in metastatic CRC.

## Data Availability

The mRNA-seq data generated in this study have been deposited in GEO under accession GSE305642.

## Supplementary Materials

The following supporting information can be downloaded at: https://www.mdpi.com/article/10.3390/cells15010029/s1. Table S1. ShRNA sequences. Table S2. siRNA sequences. Table S3. The primer sequences for qPCR. Table S4. Details of antibodies and reagents. Table S5. Correlation between TIMP1 expression and clinicopathologic features. Table S6. The prognostic significance of TIMP1 and clinicopathological characteristics in CRC. Supplemental Figure S1. Quality control, differential expression, and clustering analyses of GEO colorectal cancer datasets. Supplemental Figure S2. Validation of TIMP1 knockdown and overexpression in colorectal cancer cell lines. Supplementary Figure S3. Recombinant TIMP1–treated macrophages promote colorectal cancer liver metastasis in vivo. Supplemental Figure S4. Flow cytometry gating strategy for isolation of murine hepatic macrophage subsets. Supplementary Figure S5. Pharmacologic inhibition of integrin–AKT/mTOR signaling attenuates TIMP1-induced M2 macrophage polarization.
