# Supplementary material for: Integrin-Mediated TIMP1 Signaling Reprograms Liver Macrophages and Accelerates Colorectal Cancer Metastasis
Source: Cells. 2025 Dec 23;15(1):29. doi: 10.3390/cells15010029 (PMC12785128; doi:10.3390/cells15010029)
Supplement: Supplementary file 1 [file cells-15-00029-s001.zip › cells-3969755-supplementary.pdf]

Supporting Information

*Corresponding author

Zhaoxu Zheng: Department of Colorectal Surgery, National Cancer Center/National Clinical Research Center for Cancer/ Cancer Hospital, Chinese Academy of Medical Sciences and Peking Union Medical College, Beijing, China, Email: zzx_20003@cicams.ac.cn.

Guoxin Li: Cancer Center, Beijing Tsinghua Changgung Hospital, School of Clinical Medicine, Tsinghua Medicine, Tsinghua University, Beijing, PR China, Email: lgxa04510@btch.edu.cn

Supplemental Materials and Methods

TABLE S1. ShRNA sequences:

Name	sequences (5'-3')
ShNC	GCATTACGCGGATAACTCA
Sh-TIMP1-1	CCTGGTCATAAGGGCTAAATT
Sh-TIMP1-2	CCACCTTATACCAGCGTTATA

TABLE S2. siRNA sequences:

Name	Sense sequences(5'-3')	Antisense sequences(5'-3')
siNC	UUCUCCGAACGUGUCACG UTT	ACGUGACACGUUCGGAGAATT
siCD63-1	GGGAAUGAUUUCAAGGAAUUU	AUUCCUUGAAAUCAUUCCCUU
siCD63-2	GCAAUAUGGCUAAGGAAGAUU	UCUUCCUUAGCCAUAUUGCUU
siCD63-3	CGUGGAGACUAUAGCAAUAUU	UAUUGCUAUAGUCUCCACGUU
siβ1-integrin-1	GACUAUUGAAAUCAAGCUUAUUGGA	UCCAAUAAGCUUGAUUUCAAUAGUC
siβ1-integrin-2	AGUGAAGACAUGGAUGCUUACUGCA	UGCAGUAAGCAUCCAUGUCUUCACU

TABLE S3. The primer sequences for qPCR were performed as follows:

Name	Forward primer (5'-3')	Reverse primer (5'-3')
TIMP1	TTGGCTGTGAGGAATGCACA	GTCCACAAGCAATGAGTGCC
Timp1	CCAGAACCGCAGTGAAGAGT	GGACCTGATCCGTCCACAAA
GAPDH	AATGGGCAGCCGTTAGGAAA	GCCCAATACGACCAAATCAGAG
Gapdh	ACCCTTAAGAGGGATGCTGC	CCCAATACGGCCAAATCCGT
Csf1	GCCCTTCTTCGACATGGCT	GTTCTGACACCTCCTTGGCA
Irf4	TCTGCCAGCCCAGGTTCATA	CAGGTGGGGCACAAGCATAA
Cd36	TGTGGAGCAACTGGTGGATG	CGTGGCCCGGTTCTAATTCA
Plat	CAAGCACTCTCGGGACACAG	TTGTCTGCGTTGGCTCATCT
Tlr2	GAGCATCCGAATTGCATCACC	TCCTCTGAGATTTGACGCTTT
Tlr13	ACAGTGAATGGTGCCGACTT	AACGCATTTCTGATGCGAGC
Nfkbid	AGTCTGCATCCAAGAATCCTC	AAATGAGTATGGCCTGGCTCT

TABLE S4. Details of antibodies and reagents are as follows:

**Reagent or Resource**	**Source**	**Identifier**
**Antibodies**		
GAPDH	Proteintech	Cat. No. 10494-1-AP
TIMP1	Cell Signaling Technology	Cat. No. 8946
CD206	Cell Signaling Technology	Cat. No. 24595
CD163	Cell Signaling Technology	Cat. No. 93498
Phospho-PRAS40 (Thr246)	Cell Signaling Technology	Cat. No. 2997
PRAS40	Cell Signaling Technology	Cat. No. 2691
Phospho-mTOR(Ser2448)	Cell Signaling Technology	Cat. No. 2971
mTOR	Cell Signaling Technology	Cat. No. 2972
Phospho-AKT	ABclonal Technology	Cat. No. AP0982
AKT	ABclonal Technology	Cat. No. A20799
InVivoMAb anti-mouse Ly6G	Bio X Cell	Cat. No. BE0075
InVivoPlus anti-mouse NK1.1	Bio X Cell	Cat. No. BP0036
Anti-Rabbit IgG	Proteintech	Cat. No. SA00001-2
Anti-Mouse IgG	Cell Signaling Technology	Cat. No. 7076P2
Clodronate Liposomes	MCE	HY-172202
Cilengitide	MCE	HY-16141
Human TIMP1 Recombinant Protein	ThermoFisher Scientific	Cat. No. RP-75735
Mouse TIMP1 Recombinant Protein	Abcam	Cat. No. ab206786
Recombinant Human IL-4 protein	Abcam	Cat. No. ab119465
Recombinant mouse IL-4 protein	Abcam	Cat. No. ab259406
Recombinant Human IL-13 protein	Abcam	Cat. No. ab270079
Recombinant mouse IL-13	Abcam	Cat. No. ab270080

protein

TABLE S5. Correlation between TIMP1 expression and clinicopathologic features (n = 110).

Clinicopathologic features	All patients	Expression of TIMP1		p-Value
		Low (n = 58)	High (n = 52)	
Gender				0.110
Female	49	30	19	
Male	61	28	33	
Age, years				0.068
<60	39	16	23	
≥60	71	42	29	
Tumour size, cm				0.515
<5	62	31	31	
≥5	48	27	21	
Tumour infiltration				0.220
T1 + T2	24	10	14	
T3 + T4	86	48	38	
Lymph node metastasis				0.043
No	72	43	29	
Yes	38	15	23	
Distant metastasis				0.008
No	104	58	46	
Yes	6	0	6	
TNM stage				0.105

I + II	72	42	30
III + IV	38	16	22

TABLE S6. The prognostic significance of TIMP1 and clinicopathological characteristics in CRC.

Clinicopathologic features	Univariate analysis		Multivariate analysis	
	5-year OS	p[a]	HR	p[b]
Gender				
Female	84.4	0.283		
Male	76.8			
Age, years				
<60	86.3	0.109		
≥60	79.0			
Tumour size, cm				
<5	80.7	0.317		
≥5	82.6			
Tumour infiltration				
T1 + T2	86.5	0.34		
T3 + T4	80.3			
Lymph node metastasis				
No	89.6	0.019	1	
Yes	66.0		2.154	0.068
Distant metastasis				

No	85.8	<0.001	1	
Yes	0		5.424	0.002
TNM stage				
I + II	88.1	0.071		
III + IV	66.8			
TIMP1				
Low	90.8	0.004	1	
High	61.9		4.644	**0.007**

TIMP1 tissue inhibitor of metalloproteinase 1, CRC colorectal cancer, OS overall survival, HR hazard ratio.

a Calculated by log-rank test.

b Calculated by Cox-regression Hazard model.

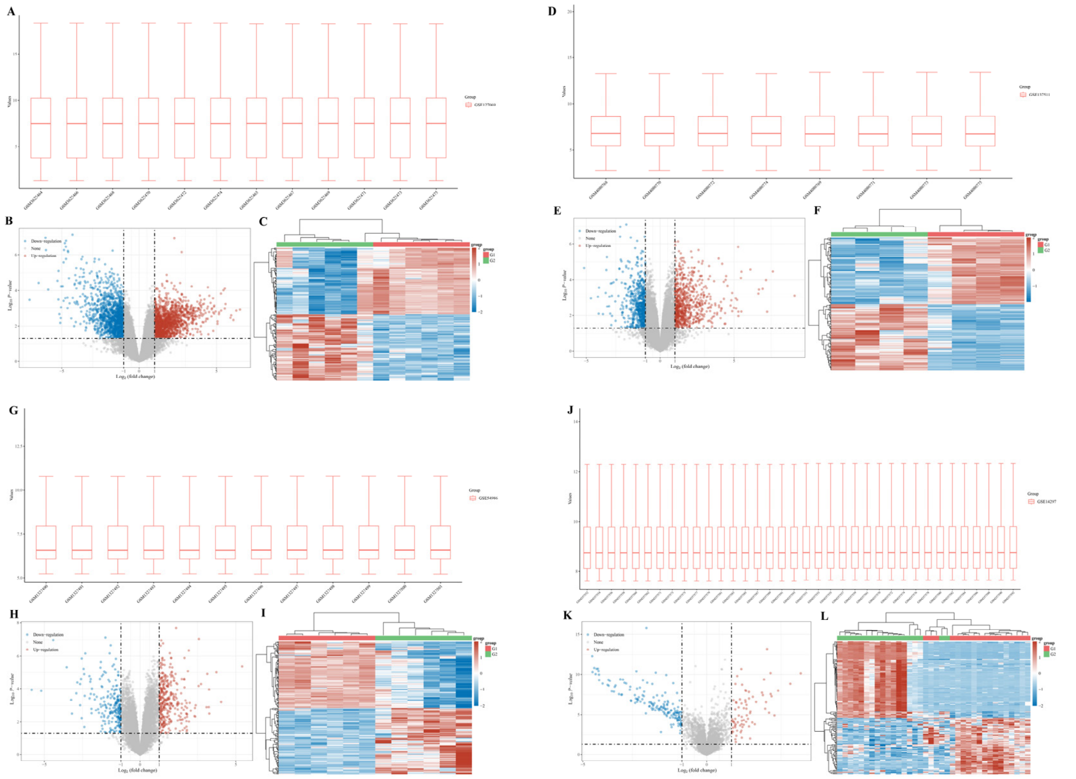

Supplemental Figure S1. Quality control, differential expression, and clustering analyses of GEO colorectal cancer datasets

(A–C) Analysis of GSE127069: (A) Boxplots showing normalized gene expression distributions across tumor and normal samples. (B) Volcano plot displaying significantly upregulated (red) and downregulated (blue) genes. (C) Heatmap of differentially expressed genes (DEGs) showing distinct separation of tumor and normal tissues.

(D–F) Analysis of GSE137511: (D) Boxplots of normalized expression data. (E) Volcano plot of DEGs. (F) Heatmap of DEGs.

(G–I) Analysis of GSE54986: (G) Boxplots of normalized expression data. (H) Volcano plot of DEGs. (I) Heatmap of DEGs.

(J–L) Analysis of GSE14297: (J) Boxplots of normalized expression data. (K) Volcano plot of DEGs. (L) Heatmap of DEGs.

All DEGs were identified using the thresholds of |log2 fold change| > 1 and adjusted p < 0.05. Red and blue colors in volcano plots represent significantly upregulated and downregulated genes, respectively. Heatmaps were generated by unsupervised

hierarchical clustering, with red indicating higher and blue indicating lower relative expression levels.

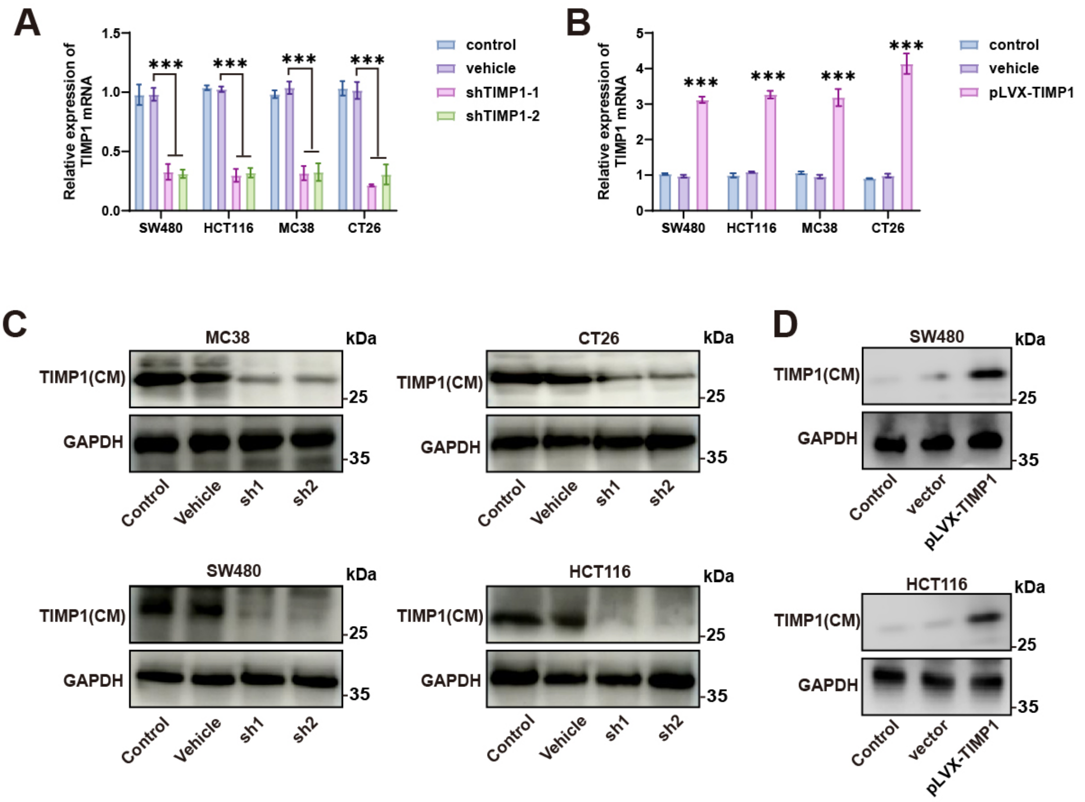

Supplemental Figure S2. Validation of TIMP1 knockdown and overexpression in colorectal cancer cell lines

(A) qPCR analysis of TIMP1 mRNA levels in human (SW480, HCT116) and murine (MC38, CT26) CRC cells transduced with shTIMP1-1, shTIMP1-2, or control vectors.

(B) qPCR analysis of TIMP1 mRNA expression in CRC cells following lentiviral overexpression (pLVX-TIMP1) compared with vehicle and control groups.

(C) Western blot detection of TIMP1 protein in conditioned media (CM) from murine (MC38, CT26) and human (SW480, HCT116) CRC cells after transduction with control, vehicle, shTIMP1-1, or shTIMP1-2.

(D) Western blot detection of TIMP1 protein in conditioned media (CM) from SW480 and HCT116 cells under control, vector, or pLVX-TIMP1 overexpression conditions. GAPDH was used as a loading control.

Data are presented as mean ± SEM from three independent experiments. Statistical analysis was performed using one-way ANOVA followed by post-hoc tests. ***p <

0.001.

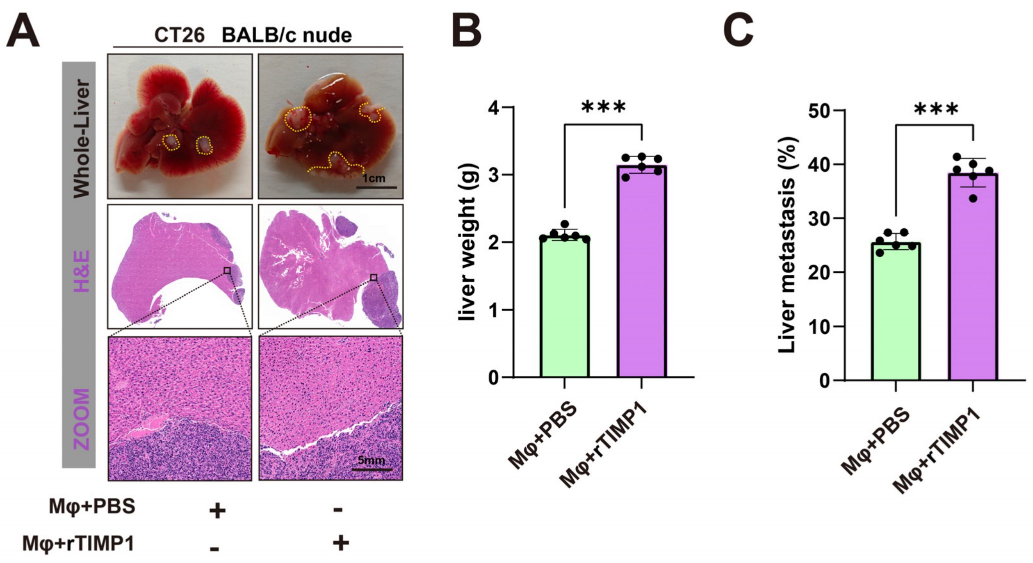

Supplementary Figure S3. Recombinant TIMP1–treated macrophages promote colorectal cancer liver metastasis in vivo

(A) Representative gross liver images and H&E staining from BALB/c nude mice co-injected intrasplenically with CT26 cells and PBS-treated macrophages (Mφ+PBS) or recombinant TIMP1–treated macrophages (Mφ+rTIMP1). Yellow dotted lines indicate metastatic lesions. Scale bars: whole-liver, 1 cm; zoom, 5 mm. (B) Quantification of liver weight. (C) Percentage of liver surface occupied by metastatic lesions. Data are presented as mean ± SEM (n = 6 mice per group). ***$p < 0.001$ by unpaired two-tailed Student's t-test.

A

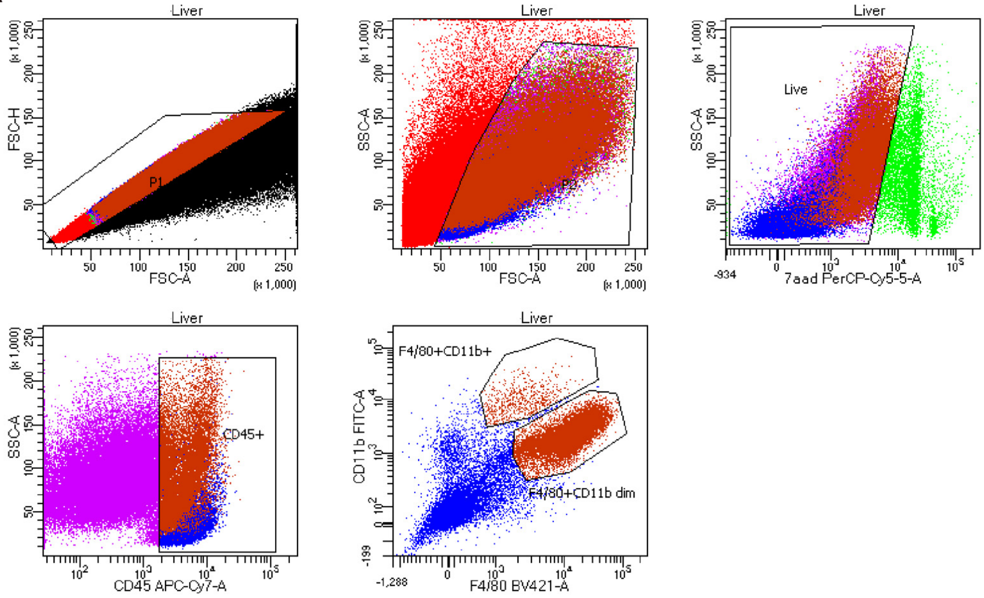

Tube: Liver

Population	#Events	%Parent	%Total
All Events	1,602,119	####	100.0
P1	1,061,345	66.2	66.2
P2	334,761	31.5	20.9
Live	306,227	91.5	19.1
CD45+	101,120	33.0	6.3
F4/80+CD11b dim	44,587	44.1	2.8
F4/80+CD11b+	2,586	2.6	0.2
P5 OR P6(sort)	47,173	46.7	2.9

Supplemental Figure S4. Flow cytometry gating strategy for isolation of murine hepatic macrophage subsets

(A) FACS gating strategy for isolation of murine hepatic macrophages. Representative plots from BALB/c liver single-cell suspensions. P1: singlets (FSC-H vs FSC-A). P2: forward/side-scatter (FSC-A vs SSC-A) to exclude debris. Live: 7-AAD– viable cells (7-AAD, PerCP-Cy5.5). CD45+: leukocytes (CD45, APC-Cy7). Final bivariate gating of F4/80 (BV421) versus CD11b (FITC) resolves F4/80^+CD11b^dim (Kupffer-cell–enriched) and F4/80^+CD11b^+ (monocyte-derived macrophage–enriched) subsets. Numbers in the table denote percentages relative to the parent gate. One representative experiment is shown.

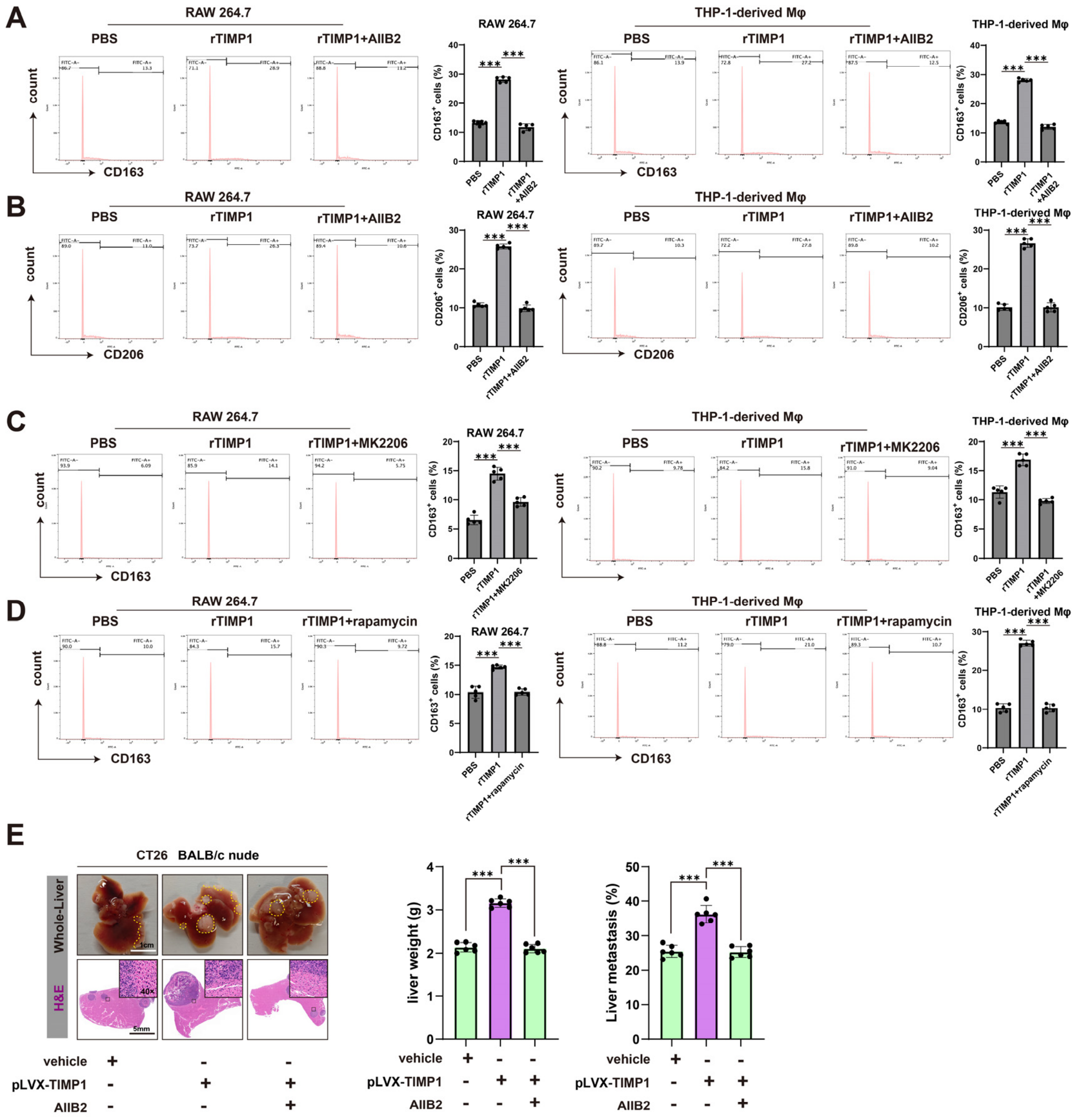

Supplementary Figure S5. Pharmacologic inhibition of integrin–AKT/mTOR signaling attenuates TIMP1-induced M2 macrophage polarization

(A–B) Flow cytometry analysis of CD163$^+$ and CD206$^+$ cells among RAW264.7 and PMA-differentiated THP-1 macrophages treated with PBS, recombinant TIMP1 (rTIMP1, 100 ng/mL), or rTIMP1 in the presence of the β1-integrin–blocking antibody AIIB2 (10 μg/mL). Cells were pre-incubated with AIIB2 for 1 h, followed by co-treatment with rTIMP1 for 24 h before staining.

(C) Flow cytometry analysis of CD163$^+$ cells in RAW264.7 and THP-1–derived macrophages treated with PBS, rTIMP1, or rTIMP1 plus the AKT inhibitor MK2206 (1 μM). MK2206 was added 1 h before rTIMP1 and maintained during the 24h

incubation.

(D) Flow cytometry analysis of $CD163^{+}$ cells in RAW264.7 and THP-1–derived macrophages treated with PBS, rTIMP1, or rTIMP1 plus the mTOR inhibitor rapamycin (20 nM; pre-treatment 1 h, then co-incubation with rTIMP1 for 24 h).

(E) Representative liver photographs, H&E staining, liver weights, and percentage of liver metastases in BALB/c nude mice injected intrasplenically with CT26 cells overexpressing TIMP1 (pLVX-TIMP1) and treated with vehicle or the β1-integrin–blocking antibody AIIB2 (10 mg/kg, intraperitoneally, on days 0, 3, and 6 after tumor cell injection). Data are shown as mean ± SEM (n = 6 per group). Statistical significance was determined by one-way ANOVA followed by post-hoc tests; ***$p < 0.001$.
